# Topological Data Analysis Combined with High-Throughput Computational Screening of Hydrophobic Metal–Organic Frameworks: Application to the Adsorptive Separation of C3 Components

**DOI:** 10.3390/nano14030298

**Published:** 2024-01-31

**Authors:** Yujuan Yang, Shuya Guo, Shuhua Li, Yufang Wu, Zhiwei Qiao

**Affiliations:** Guangzhou Key Laboratory for New Energy and Green Catalysis, School of Chemistry and Chemical Engineering, Guangzhou University, Guangzhou 510006, China; 2112105114@e.gzhu.edu.cn (Y.Y.); 2112105073@e.gzhu.edu.cn (S.G.); lish@gzhu.edu.cn (S.L.)

**Keywords:** topological data analysis (TDA), metal–organic frameworks (MOFs), topology of pores, petroleum, adsorption

## Abstract

The shape and topology of pores have significant impacts on the gas storage properties of nanoporous materials. Metal–organic frameworks (MOFs) are ideal materials with which to tailor to the needs of specific applications, due to properties such as their tunable structure and high specific surface area. It is, therefore, particularly important to develop descriptors that accurately identify the topological features of MOF pores. In this work, a topological data analysis method was used to develop a topological descriptor, based on the pore topology, which was combined with the Extreme Gradient Boosting (XGBoost) algorithm to predict the adsorption performance of MOFs for methane/ethane/propane. The final results show that this descriptor can accurately predict the performance of MOFs, and the introduction of the topological descriptor also significantly improves the accuracy of the model, resulting in an increase of up to 17.55% in the *R*^2^ value of the model and a decrease of up to 46.1% in the RMSE, compared to commonly used models that are based on the structural descriptor. The results of this study contribute to a deeper understanding of the relationship between the performance and structure of MOFs and provide useful guidelines and strategies for the design of high-performance separation materials.

## 1. Introduction

In nanoporous materials, the pore structure has a significant impact on the performance of the material, which can affect the strength, thermal conductivity, adsorption capacity, and other key properties of the material. By adjusting the pore size, distribution, and shape, the functionality and performance of the material can be precisely modulated. Yongjin [1] found that the performance of porous materials for carbon capture or methane storage could be improved by several orders of magnitude simply by modifying the pore structure. Conventional porous materials, such as zeolites and activated carbons, have applications in separation and adsorption, but their pore structures are usually fixed. In contrast, metal–organic frameworks (MOFs), which are composed of organic ligands and metal clusters, have highly tunable pore structures. MOFs represent a class of porous materials composed of metal ions or clusters combined with organic ligands, and their high degree of tunability and excellent pore structures have made them highly interesting, cutting-edge materials in the field of gas separation. The unique properties and versatility of MOF materials offer great potential for applications in natural gas separation [2] and storage [3], as well as in gas adsorption [4], separation [5], and transport [6].

The rapid development of the global economy and population growth have led to an increase in energy consumption, resulting in a sharp rise in the emissions of greenhouse gases and pollutants. Countries all over the world have been at the forefront of efforts to mitigate this problem. For instance, in China, the government strongly advocates for low-carbon and green energy, leading to increased attention and investment in the natural gas sector. In recent years, natural gas production and consumption have shown strong upward trends [7]. Clean and efficient natural gas is currently considered one of the most strategic options for optimizing energy structures, saving energy, and reducing emissions. However, natural gas typically contains a range of hydrocarbon components with different carbon chain lengths: methane (C1) typically makes up 70 to 90 percent of natural gas, while ethane (C2) and propane (C3) are also important components, with contents ranging from 0 to 22 percent. These components are critical for energy supply, chemical production, and industrial applications. Methane is an essential component of natural gas for transportation and storage and is widely used for domestic and industrial heating, and as a fuel. Pure ethane, on the other hand, is an important raw material in the petrochemical industry, used in the synthesis of ethylene and other chemicals, including plastics and synthetic rubber. Pure propane can be used to produce liquefied petroleum gas (LPG) and is also used in the synthesis of chemicals, such as propylene and propylene glycol. Efficient separation of the C1/C2/C3 components, which is recognized as one of the seven separation processes that have had a significant impact on the world [8], is critical to the production, processing, and transportation of natural gas. Conventional methods for separating C1, C2, and C3 face several challenges [9,10]. Firstly, conventional distillation and adsorption methods require high temperatures and pressure, resulting in increased energy consumption, costs, and greenhouse gas emissions. Secondly, due to the high similarity among the components, conventional methods have limited separation efficiency, making it difficult to achieve high-purity separation. However, using adsorption separation technology [11,12] to separate C1, C2, and C3 offers several advantages, including high selectivity, renewability, controllability, and environmental friendliness. Chen’s group [13,14,15] synthesized a series of MOFs, called USTA, to separate C1–C3 through adsorption; Zhang et al. [16] synthesized a hydrophobic metal–organic framework, UPC-21, using polyaromatic units, for efficient separation of C2/C1; Li’s group [17] proposed a new strategy for the synthesis of MIL-100 (Fe), facilitated by room temperature oxidizing radicals for the separation of C1–C3 ternary gas mixtures.

In recent years, machine learning (ML) has been increasingly used to screen high-performance MOFs, especially in the field of material design and discovery. Luo et al. [18] used automated data mining and machine learning models to predict the rationalization of MOF synthesis conditions, which accelerated the discovery process for MOFs; Wang et al. [19] discovered a high-performance MOF for ethane/ethylene separation using interpretable machine learning; Hakan et al. [20] performed a computational screening of MOFs for acetylene separation and found that anionic columnar MOFs exhibited high performance; Hilal et al. [21] developed a machine learning model that accurately predicted the adsorption and diffusion characteristics of six gases (He/H_2_, He/N_2_, He/CH_4_, H_2_/N_2_, H_2_/CH_4_, and N_2_/CH_4_) in MOFs. ML can rapidly analyze the structure and properties of MOFs to help screen the most promising candidates from large MOF databases, speeding up the material screening process and reducing the cost of trial and error. In addition, ML can reveal the complex relationship between the structure and properties of MOFs, automating the material design process. This can help in the discovery of new material design principles and improve the efficiency of material design, leading to new opportunities and breakthroughs in material science. In most applications of MOFs, the pore topology has as important an impact on the performance of an MOF as its chemical composition, but less research has been conducted on the relationship between pore topology and the performance of MOFs. 

In this work, we developed a topological descriptor, based on the persistence barcodes of MOF pore structures, which are representations of the pore topological information of MOFs, obtained through topological data analysis techniques, and constructed an automated processing software, which can automatically generate the topological descriptor based on the crystallographic information file (.cif) of MOFs. Then, we predicted the performance of MOFs and investigated the performance of the machine learning model with different combinations of descriptors. The topological descriptor was found to have a more important influence in predicting the performance of materials. Finally, the feature importance of different target gas adsorption predictions was analyzed by combining the machine learning feature importance method.

## 2. Model and Methods

### 2.1. Molecular Model

In this study, we used a large crystallographic dataset of 137,953 hMOFs, as derived by Wilmer et al. [22]. To eliminate the influence of highly hydrophilic hMOFs, we screened 31,399 hydrophobic hMOFs, based on their Henry coefficients of water vapor. We then used high-throughput molecular simulations to calculate the structural descriptors of the MOFs, which included their porosity (*φ*), density (*ρ*), volumetric surface area (VSA), and largest cavity diameter (LCD). The LCDs were calculated using Zeo++ version 0.3 software [23]. The VSA and *φ* were calculated using RASPA version 1.9.15 software [24], using He with a diameter of 2.58 Å and N_2_ with a diameter of 3.64 Å as probe molecules. The N_2_ model is an uncharged spherical model.

The interaction between the adsorbate atoms and the MOF was described using the Lennard-Jones (LJ) and electrostatic potentials:

(1) $$u_{LJ+elec}\left(r\right)=\sum 4\varepsilon_{ij}\left[{\left(\frac{\sigma_{ij}}{r_{ij}}\right)}^{12}-{\left(\frac{\sigma_{ij}}{r_{ij}}\right)}^{6}\right]+\sum \frac{q_{i}q_{j}}{{4\pi \varepsilon }_{0}r_{ij}}$$

where $u_{\mathrm{LJ}+\mathrm{elec}}(r)$ is the interaction potential energy between atom *i* and atom *j*; $\varepsilon_{ij}$ and $\sigma_{ij}$ denote the depth of the potential energy and the location where the LJ potential energy is zero (also known as the point of contact), respectively; $r_{ij}$ denotes the distance between the interacting atoms; $\sigma_{ij}$ represents the equilibrium distance between the atoms; $q_{i}$ and $q_{j}$ denote the atoms’ charges; $\varepsilon_{0}=8.8542\;\times \;10^{-12}C^{2}\cdot N^{-1}$ denotes the vacuum permittivity. The LJ potential energy parameters of all hMOFs come from the Universal Force Field (UFF) [25], as shown in Table S1 (Supplementary Materials). The atomic charges of MOFs were calculated using the MEPO-Qeq method.

### 2.2. GCMC Simulation

In this work, GCMC simulations of the adsorption properties of C1, C2, and C3 gas mixtures in natural gas were calculated by simulating hMOFs at 298 K and 1 × 10^6^ Pa, for which the ratio of the amount of substance of the ternary gas mixtures C1, C2, and C3 was 7:2:1. The RASPA package was used for the simulation process, and each MOF was simulated independently. The MOF structure remained rigid throughout each simulation. The interactions between the MOFs and the gas mixture were calculated using the Lorentz-Berthelot rule. The cells were simulated with periodic boundary conditions along each direction, extended to at least 24 Å in the *x*, *y*, and *z* directions. LJ interactions were calculated by setting the spherical truncation radius to 12 Å. The Ewald summation method was employed for electrostatic interactions in the calculation of the Henry’s coefficient for water. Electrostatic interactions were calculated using the Ewald summation method. All GCMC simulations were performed using the RASPA software. The simulations were conducted for 200,000 cycles for each MOF. The first 100,000 cycles were used for equilibration, and the last 100,000 cycles were used for ensemble averages. Each cycle comprised n GCMC experimental moves (where n is the number of adsorbate molecules). The GCMC moves included translation, rotation, regeneration, and exchange. The simulation’s accuracy was also verified by testing different numbers of GCMC cycles. It was discovered that increasing the number of cycles had little impact on the simulation results.

### 2.3. Datamining the Topology of MOF Pores

#### 2.3.1. Topological Data Analysis

MOF crystals possess unique pore topology. To analyze the topological features of MOFs with large amounts of data, we use Topological Data Analysis (TDA), which employs persistent homology [26] to calculate the topological features of data at different scales. The main objective is to record the topological invariants of the structure, such as *β*_0_, *β*_1_, and *β*_2_, as the atomic scale changes. These invariants are denoted as persistent barcodes. The persistent barcodes generated correspond to topological features such as connected components, holes, and higher dimensional counterparts (e.g., cavities) in the data. Professors Pan and Wei [27] have introduced a mathematical method into material science that utilizes persistent homology. This method maps material structures from high-dimensional space to low-dimensional topological space, thus accelerating the study of the relationship between topology and material properties.

#### 2.3.2. Persistent Homology

Persistent homology is widely used to understand features in data, especially when dealing with complex structures and multi-scale data. Persistent homology [28,29] is a technique used in topological data analysis to capture topological structures in a dataset and measure their persistence or stability at different scales. As the filtering radius increases, connections between points generate simplexes. These simplexes include 0-simplexes (points), 1-simplexes (line segments), 2-simplexes (triangles), 3-simplexes (tetrahedra), and so on. These simplexes combine to form higher-dimensional simplexes, and persistent homology captures the state of these composites at different filtering radii. In homology theory, a homology group is an algebraic structure used to characterize the topology of a space. The homology groups’ dimensions and generators provide information on the topological structures present in the dataset, such as connected components, holes, and voids in space. The homology group H_k_(X) is represented by an abstract generating element that reflects the k-dimensional topology. The dimension b_k_(X) (i.e., *β*_k_) indicates the number of linearly independent generating elements in the k-dimensional homology group. For example, in the case of the 0-dimensional homology group, the generating element is the connected component, and the dimension is the number of connected components. For each homology group, we can visualize how the homology features vary with the filter radius by constructing a persistence barcode. The horizontal axis in the persistence barcode represents the radius. The bar represents the process, from birth to death, of a topological feature, and the length of the bar (death–birth) represents the persistence of this topological feature.

#### 2.3.3. Pore Topology Persistence Barcode

**Figure 1:**
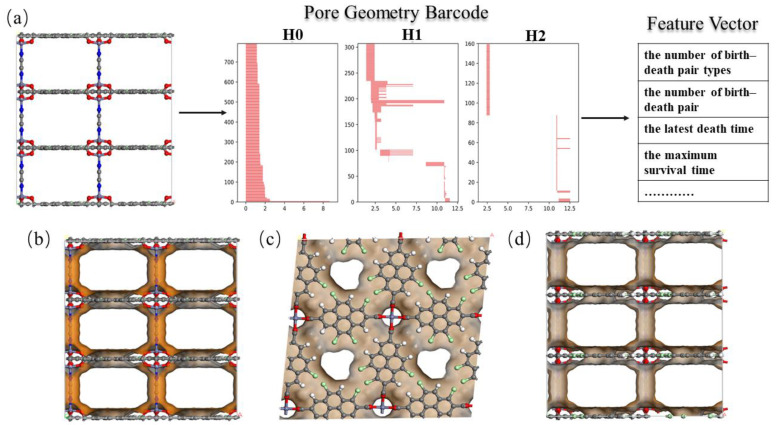
(**a**) Flowchart of the development of the topological descriptor for hMOF5035530 (the horizontal coordinate in the pore geometry barcode represents the filter radius and the vertical coordinate represents the number of barcodes). (**b**–**d**) Structure of hMOF5035530. Red balls represent oxygen atoms, grey balls represent carbon atoms, white balls represent hydrogen atoms, green balls represent chlorine atoms, blue balls represent nitrogen atoms and grey-blue balls represent zinc atoms.

Persistence barcodes are used to characterize the pore structures of materials by encoding information about the pore structure of MOFs into the unique form of data. They were used to data-materialize the topology of the MOFs, and they can be used as descriptors to provide representations of the pore structures of MOFs. To obtain the persistence barcode for each MOF, the atomic coordinates were first acquired using the pymatgen [30] tool, and then the persistence barcode was computed by inputting the atomic coordinates into the persistence homology point cloud of the topology machine learning tool giotto-tda [31]. Finally, the homology group and connectivity number of each MOF were output in the dimensions corresponding to *β*_0_, *β*_1_, and *β*_2_ for isolated components, holes, and cavities (Figure 1a).

During the persistent homology computation, point-to-point connections lead to the birth and death of topological features as the distance parameter increases, and each topological feature is assigned a birth time and a death time; features with longer durations (death–birth) are usually of significant relevance, while features with shorter persistence durations are usually considered noise. Figure 1b–d show the pore structure of hMOF5035530, in which the 1D long-spaced barcodes in Figure 1a represent the number of channels in the pore system of the MOFs, and the 2D long-spaced barcodes represent the number of connecting cavities between the lamellar channels. It is often important to track the birth, death, and duration of each barcode, as this information is related to the bond lengths, rings (or channels), and cavity sizes of the unique structures in the MOFs. To extract features from the generated material barcodes and obtain vectorized feature vectors, we counted the number of minimums, maximum, mean, standard deviation, and sum, as well as the birth–death pairs of birth, death, and persistence information for each barcode in different dimensions. Thus, for each MOF, we have a total of 42 topological representations specific to its pore structure, as shown in Table S2 (Supplementary Materials). These descriptors capture the structural topological information of the MOF materials, including the pore structure, the type of connectivity, and the location of the functionalized groups, and they are able to quantitatively materialize the structural features of the MOF materials.

## 3. Results and Discussion

### 3.1. Structure–Performance Relationships

**Figure 2:**
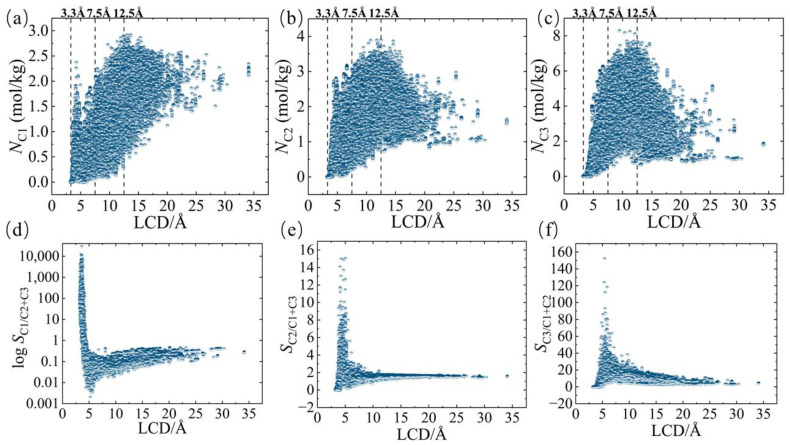
Relationships between (**a**) *N*_C1_ and LCD, (**b**) *N*_C2_ and LCD, (**c**) *N*_C3_ and LCD, (**d**) $\log S_{C1/C2+C3}$ and LCD, (**e**) *S*_C2/C1+C3_ and LCD, and (**f**) *S*_C3/C1+C2_ and LCD.

Following the high-throughput calculations, univariate analyses were conducted to investigate the correlation between the material structure and the separation performance of the complex ternary gas mixtures of C1, C2, and C3 in the MOFs. Figure 2a–c illustrate the relationship between the adsorption of C1, C2, and C3 components and LCD. When LCD is less than 2.5 Å, the adsorption of alkanes by MOFs is limited by the space between alkane molecules and pore walls, resulting in almost no adsorption. When the length of the shortest distance between the alkane molecules and the skeleton molecules of MOFs is between 2.5 Å and 6 Å, the intermolecular relative force increases with the length of the shortest distance, and the amount of adsorption also increases. A peak appears, known as the first peak, corresponding to the LCD, which is slightly larger than the kinetic diameter of the C1/C2/C3 molecule (C1~3.8 Å, C2~3.9 Å, and C3~4.3 Å). As the LCD increases, the interaction between the backbone molecules and the alkane molecules weakens, causing *N*_C1_–*N*_C3_ to decrease. Surprisingly, the adsorption increases again when the LCD is between 6.5 Å and 12.5 Å, forming a relatively strong peak known as the second peak. The second peak corresponds to approximately three times the kinetic diameter of the C1/C2/C3 molecule. The intensities of the first and second peaks gradually increase with the growth of the carbon chain. In the univariate analysis, we analyzed the relationships between the selectivity of individual components C1, C2, and C3 and LCD. Log transformations were applied to reduce the effects of extreme values due to the wide distribution of the variable *S*_C1/C2+C3_. Figure 2d–e show the relationships between the selectivity of C1, C2, and C3 components and LCD. The selectivity distribution plots of C1, C2, and C3 exhibit significant peaks at different values of LCD, corresponding to the kinetic diameter of the C1/C2/C3 molecule. This peak gradually shifts backward with the growth of the carbon chain. The selectivity peak appears at smaller LCD values because the molecular radii of C1, C2, and C3 gradually increase, and C1 molecules with shorter carbon chains can enter smaller pores more easily. As the carbon chain length increases, the molecules of C2 and C3 require larger pores to be adsorbed efficiently, causing the selectivity peaks to shift gradually back to larger LCD values. The discovery of the second peak in the constitutive analysis is similar to the work of Yuan et al. [32] and effectively broadens the scope of the structural design of high-performance materials. 

### 3.2. Machine Learning

In order to analyze the behaviors of MOFs for the separation of ternary C1, C2, and C3 gas mixtures and to evaluate their overall performance, we have introduced the variable TSN (trade-off between *S*_C3/C1+C2_ and *N*_C3_) as an adsorption-selectivity trade-off variable, which was previously used by Shah et al. [33] to evaluate the performance of molecular sieve adsorbents for the removal of H_2_S. The formula for calculating TSN is as follows:

(2) $$\mathrm{TSN}=N_{i}\times \ln S_{\frac{i}{j_{1}+j_{2}}}$$

We added the topological and structural descriptors as descriptors to predict the adsorption separation performance of MOFs against a ternary gas mixture of C1/C2/C3. To test the accuracy, robustness, and efficiency of the topological features, we employed the XGBoost [34] algorithm. This integrated learning algorithm is an extension of the gradient boosting algorithm, which improves the model’s generalization ability and prediction accuracy by integrating multiple weak learners. The XGBoost algorithm simplifies the model by using the regular term technique to avoid overfitting. The hyperparameters used in XGBoost are detailed in the Supplementary Materials. Chen et al. [35] also considered XGBoost to be the optimal method for predicting the MOFs’ adsorption systems. The ML model was constructed using scikit-learn version 1.2.1 [36] software. In this work, data from 31,399 hydrophobic hMOFs were divided into training and testing sets in the ratio of 8:2. The model was evaluated using the root mean square error (RMSE) and the coefficient of determination (*R*^2^), as shown in Supplementary Materials.

**Figure 3:**
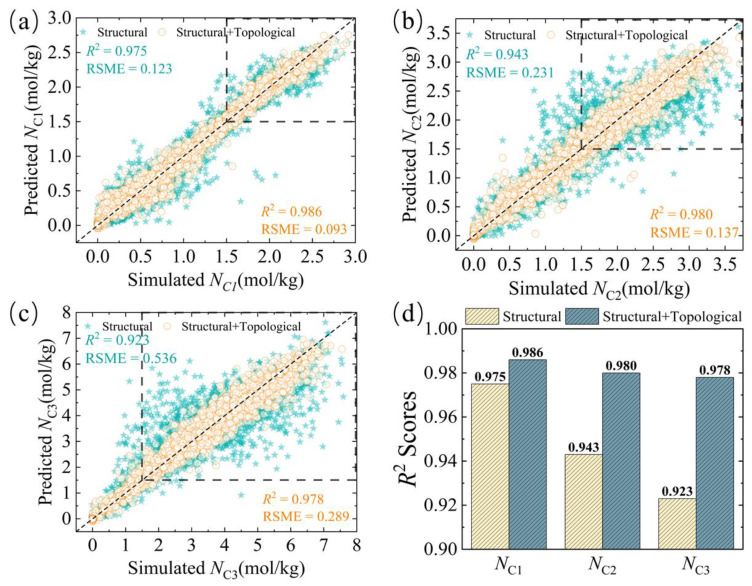
Distribution of simulated and predicted data for (**a**) *N*_C1_, (**b**) *N*_C2_, and (**c**) *N*_C3_. (**d**) *R*^2^ scores for models using different feature sets in different systems.

The accuracy and versatility of the topological descriptors were evaluated by using the XGBoost algorithm to predict the *N*, *S*, and TSN of C1, C2, and C3. The results of the models trained with the structural descriptors, topological descriptors, and the combination of these descriptors (S + T) were computed for different target gases (Table 1 and Table S3 (Supplementary Materials)). (i) the addition of topological descriptors helps to improve the overall model performance and reduce model-related errors, but the selective holistic models for C1 and C2 show poor prediction results (*R*^2^ < 0.67). (ii) The model shows a general prediction (*R*^2^ > 0.80) for the *S*_C3_, which can be attributed to the fact that the topological descriptors more accurately capture the pore geometry of the MOFs, thus providing a better explanation and prediction of the adsorption behavior of C3 long-chain molecules. (iii) The overall model demonstrates improved prediction for C1, C2, and C3 adsorption quantities. The *R*^2^ value of the model exceeds 0.88 when using only the structural or topological descriptor. When combining S + T descriptors, the *R*^2^ value of the model exceeds 0.97 (as shown in Figure 3d), which suggests that S + T play a synergistic role in the prediction of adsorption quantities, and improve the performance of the model because the topological descriptor records the sizes of all channels in the MOFs, as well as the size information of different cavities, which can capture information not contained in the structural descriptor. Topological descriptors can capture multi-scale geometrical information of the material, enabling the model to consider the multi-faceted features of the molecular structure comprehensively [37]. (iv) The prediction of TSN is highly accurate for TSN_C1_ and TSN_C3_ (*R*^2^ > 0.93), and TSN_C2_ can also achieve an *R*^2^ > 0.84. This is because the topological descriptor can capture multi-scale geometrical information of the material, enabling the model to consider the multifaceted features of the molecular structure comprehensively, resulting in more accurate TSN predictions. The use of topological descriptors makes them more appropriate for predicting the performance of the C1/C2/C3-MOFs system. Therefore, XGBoost, supplemented with the combination of S + T variables, can accurately predict the performance of the C1/C2/C3-MOF system. The findings suggest that topological descriptors are superior in predicting the performance of the C1/C2/C3-MOF system. Therefore, using the XGBoost algorithm in combination with S + T variables is an efficient and accurate strategy for predicting the system’s performance.

Figure 3a–c and Figure S1 (Supplementary Materials) display the distributions of calculated and predicted data for the XGBoost algorithm using different feature sets to predict the *N*, *S*, and TSN for C1, C2, and C3. The figure shows that, after adding the topological descriptor, the yellow points are more concentrated on the diagonal than the green points, improving the overall prediction effect, especially for the medium–high performance region (*N* greater than 1.5 mol/kg), where the prediction accuracy is significantly improved. The medium–high performance region refers to MOF materials with better adsorption and separation properties. This study aims to improve the prediction of performance parameters, as accurate prediction is critical for component separation applications in natural gas. Another key improvement is that we observe an improved convergence between the simulated and predicted data; as shown in Table 1, the *R*^2^ value of the model has increased and the RMSE value has decreased, indicating a better fit of the model to the data. This improvement results in predicted values that are closer to the actual observed values, and the model performs better. The topological descriptor of the material can provide multi-scale structural information to comprehensively describe its characteristics. This has a significant impact on the MOF-C1C2C3 system, improving the fit of the XGBoost model to actual MOF performance data, and making it more consistent with theoretical simulations. Ensuring the reliability of performance predictions on unknown materials is crucial for the credibility and practicality of our model.

### 3.3. Analysis of the Relative Importance of Features

**Figure 4:**
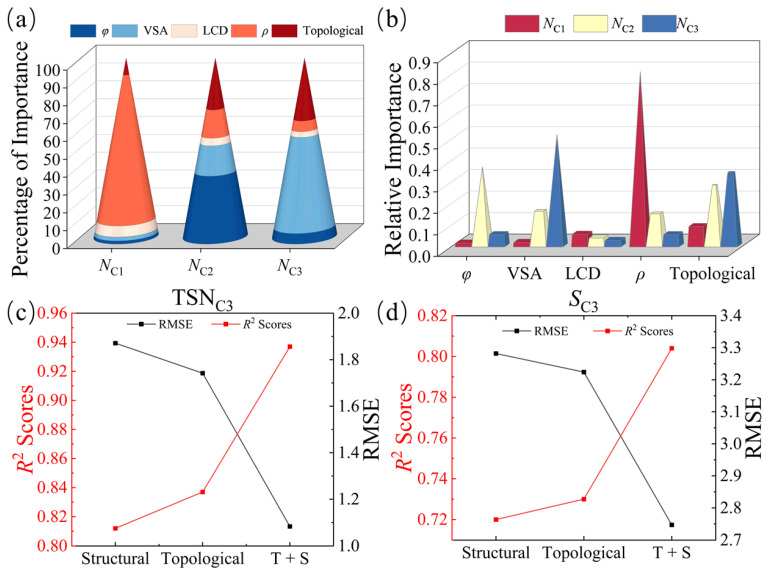
The XGBoost algorithm model predicts MOFs for different system adsorption, with (**a**) percentage of importance values and (**b**) feature importance values. Comparison of RMSE and *R*^2^ scores of the XGBoost algorithm model for predicting (**c**) TSN_C3_ and (**d**) *S*_C3_ for different feature sets.

To investigate the influences of topological descriptors on MOFs’ performance, we analyzed and quantified their relative importance using the XGBoost algorithm. In this work, the topological descriptors include three types of features: zero-dimensional features, with information such as bond lengths in the MOFs crystal structure; one-dimensional features, describing the ring and channel distributions; and two-dimensional features, describing the voids. The structural descriptors include the LCD, *ρ*, VSA, and *φ*. Figure 4a shows the relative importance values of the topological descriptors for the adsorption of the three components, C1, C2, and C3. We observe that the relative weight of the topological descriptors gradually increases as the carbon chain length increases. This suggests that MOF topological descriptors have more pronounced influences on the adsorption behavior of molecules with larger molecular sizes and longer carbon chains for the following reasons: (i) enhanced pore adaptation: as the carbon chain length increases, long-chain molecules require larger pores for effective adsorption, and the topological descriptors can capture the pore sizes, shapes, and, especially, the connectivity levels of the MOF materials to improve the prediction; (ii) with the increase in carbon chain length, the long-chain molecules will occupy more space in the structures of MOF materials, with higher contact area and stronger interaction with the pore walls, and the topological features can describe the two-dimensional spatial characteristics between MOF pores, thus reflecting the adsorption structure of long-chain molecules and improving prediction accuracy. Figure 4b shows that the topological descriptors are all the second most important descriptors in predicting the adsorption of different components, a result indicating that the topological descriptors play important roles in predicting gas adsorption. Therefore, the topological descriptors can be used to accurately predict the selective adsorption properties of C1, C2, and, especially, C3 gases, which is helpful for further screening the optimal MOFs suitable for the adsorption and separation of C3 and guiding the experimental synthesis.

**Figure 5:**
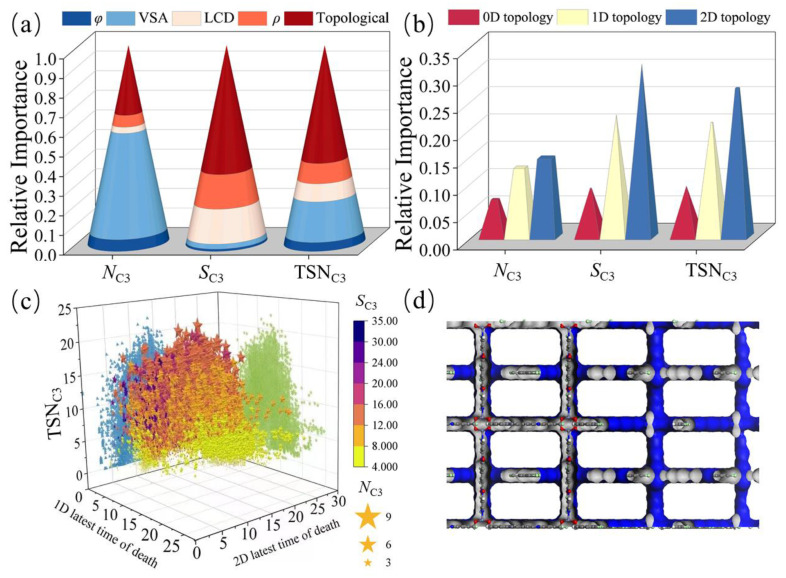
Feature importance analysis by the XGBoost algorithm model for predicting *N*_C3_, *S*_C3_, and TSN_C3_. (**a**) Relative importance values of structural descriptors and topological descriptors. (**b**) Relative importance values of topological features of different dimensions. (**c**) Relationships between 1D latest time of death, 2D latest time of death, and *N*_C3_, *S*_C3_, and TSN_C3_. The green dots represent the mapping of the graph on the yz-plane, the blue dots represent the mapping on the xz-plane, the star symbol represent the *N* of each MOF, the larger the *N*, the larger the star, and *S* is represented by the color mapping on the right of the figure. (**d**) Pore structure of hMOF5058511.

For the C1/C2/C3 components, our ML model shows excellent predictive ability, which is reflected in the prediction of *S*, *N*, and TSN, especially for long-chain C3, and for *S*_C3_ and TSN_C3_; the model performance based on the topological descriptor outperforms that based on the structural descriptor, and the combination of both performs better, as shown in Figure 4c,d. Furthermore, the effects of different topological features for *N*, *S*, and TSN of long-chain C3 are quantified and discussed, and the results are shown in Figure 5. Firstly, in this work, topological descriptors showed absolute importance in the prediction of *S_C_*_3_ and TSN_C3_ (see Figure 5a). Topological descriptors are datamined representations used to describe crystal structures, and they capture topological features of MOF crystals, such as connectivity and ring structure. These descriptors provide critical information about an MOF’s crystal structure and internal pores, which are essential for understanding adsorption properties and selectivity. Secondly, we found that, among the 42-bit topological descriptors, the 2D topological descriptors are significant in predicting *S_C_*_3_ and TSN_C3_ (see Figure 5b). Notably, the most important descriptors in the 1D and 2D topological descriptors for the prediction of TSN_C3_ were identified as the maximum death time values (21-bit and 38-bit descriptors) in the 1D and 2D topological descriptors, respectively, as shown in Figure S2 (Supplementary Materials). Data points in a persistent barcode correspond to gaps and channels of a certain size in the material. In 2D persistent barcodes, the point (b, d) is generated by the cavity of a maximum sphere, with a fitted radius of d, and the radius of the largest sphere that can enter the cavity is b. In 1D persistent barcodes, the point (b, d) reflects a one-dimensional channel in the material, specifically the narrowest ‘bottleneck’ in the channel. The d value here records the radius of the largest sphere that can pass through the bottleneck, and the b value records the minimum distance between the atoms that make up the bottleneck. These two descriptors represent the radius of the largest sphere that can enter the 2D voids and the radius of the largest sphere that can pass through the narrowest channel, respectively, which are closely related to the LCD and PLD of the MOF and play key roles in the performance of machine learning models. On this basis, we further explored the relationships between the most significant descriptors and the MOFs performance (see Figure 5c) and observed that the MOF materials exhibited significant peaks in the adsorption separation performance for C3 at values of 10~15 for the maximum death time in the 1D and 2D topological descriptors. This can be attributed to the fact that the adsorption separation of gases benefits from a more homogeneous pore structure, i.e., the closer the ratio of the maximum pore size to the minimum pore size is to 1, the more homogeneous a state the pore structure of the MOF material exhibits (see Figure 5d), which effectively facilitates the adsorption separation process of the C3 component, a result that has also been verified in a previous study [38]. This provides important guidance and insights for deepening the understanding of the factors influencing the adsorption performance of MOF materials, as well as for the rational design of efficient gas adsorption materials.

## 4. Conclusions

In this work, to further identify the pore topology of MOFs, we successfully quantified the pore topology of MOFs based on the topological data analysis method and constructed an automated processing software capable of automatically generating topological descriptors, based on the input cif files of MOF materials. For the methane/ethane/propane adsorption performance of MOFs, the XGBoost model showed accurate prediction (*R*^2^ = 0.986), indicating that the model was able to accurately predict the performance of MOFs. The results of the XGBoost feature significance analysis showed that the topological descriptors play key roles in predicting the performance of the model, and in the course of our study, we found that the relative importance of the topological descriptors gradually increased with the increase in the carbon chain length. The relative importance of the topological descriptor gradually increases, a finding that reveals an important relationship between structure and performance and provides us with a deeper understanding of MOFs. The inclusion of this descriptor significantly improves the performance of the overall machine learning model, compared to the traditional structural descriptor, and the overall performance of the topological descriptor is even significantly better than the performance of the traditional structural descriptor in the predictions of TSN_C3_ and *S_C_*_3_. The topological descriptors are generic and can be used to predict the gas adsorption properties of different systems. Our study provides insights into the relationship between the performance and structure of MOFs and offers useful guidelines and strategies for the design of high-performance separation materials. These findings not only advance the understanding of MOFs, but also provide new directions for future research in material design and application.

## Figures and Tables

**Table 1:** Evaluation of XGBoost for *N*_C1_, *N*_C2_, *N*_C3_, TSN_C1_, TSN_C2_, and TSN_C3_ (Δ represents the percentage of improvement in the model, measured by the increase in *R*^2^ and the decrease in RMSE).

Performance	*R*^2^ Scores				RMSE			
	Structural	Topological	T + S	Δ	Structural	Topological	T + S	Δ
*N*_C1_	0.975	0.896	0.986	1.13%	0.123	0.250	0.093	24.88%
*N*_C2_	0.943	0.889	0.980	3.92%	0.231	0.323	0.137	40.92%
*N*_C3_	0.923	0.885	0.978	5.96%	0.536	0.656	0.289	46.10%
TSN_C1_	0.928	0.863	0.971	4.63%	0.361	0.498	0.231	36.11%
TSN_C2_	0.718	0.715	0.844	17.55%	0.253	0.255	0.189	25.53%
TSN_C3_	0.812	0.837	0.937	15.39%	1.871	1.742	1.083	42.12%

## Data Availability

Data are contained within the article.

## Supplementary Materials

The following supporting information can be downloaded at: https://www.mdpi.com/article/10.3390/nano14030298/s1, Table S1: Lennard-Jones parameters of MOFs; Table S2: 42 Features Extracted from Barcodes; Table S3: Evaluation of XGBoost for *S*_C1_, *S*_C2_, *S*_C3_; Table S4: Lennard-Jones parameters of adsorbates; Figure S1: Distribution of computed and predicted data when the XGBoost algorithm predicts (a) *S*_C1_, (b) *S*_C2_, (c) *S*_C3_, (d) TSN_C1_, (e) TSN_C2_, and (f) TSN_C3_ using different combinations of features; Figure S2: Relative importance values of topological descriptors in the predicted TSN_C3_ (blue bars are the most important descriptors among 1D and 2D topological descriptors); Figure S3: Relative importance of the topological descriptor in predicting *S*_C3_; Figure S4: Relative importance of the topological descriptor in predicting *N*_C3_.

## References

[1] Lee Y., Barthel S.D., Dlotko P., Moosavi S.M., Hess K., Smit B. (2017). Quantifying similarity of pore-geometry in nanoporous materials. Nat. Commun..

[2] Zhou S., Shekhah O., Ramírez A., Lyu P.B., Abou-Hamad E., Jia J.T., Li J.T., Bhatt P.M., Huang Z.Y., Jiang H. (2022). Asymmetric pore windows in MOF membranes for natural gas valorization. Nature.

[3] Connolly B.M., Aragones-Anglada M., Gandara-Loe J., Danaf N.A., Lamb D.C., Mehta J.P., Vulpe D., Wuttke S., Silvestre-Albero J., Moghadam P.Z. (2019). Tuning porosity in macroscopic monolithic metal-organic frameworks for exceptional natural gas storage. Nat. Commun..

[4] Yang S.Q., Hu T.L., Chen B.L. (2023). Microporous metal-organic framework materials for efficient capture and separation of greenhouse gases. Sci. China Chem..

[5] Belmabkhout Y., Bhatt P.M., Adil K., Pillai R.S., Cadiau A., Shkurenko A., Maurin G., Liu G.P., Koros W.J., Eddaoudi M. (2018). Natural gas upgrading using a fluorinated MOF with tuned H_2_S and CO_2_ adsorption selectivity. Nat. Energy.

[6] Erdosy D.P., Wenny M.B., Cho J., DelRe C., Walter M.V., Jiménez-Angeles F., Qiao B.F., Sanchez R., Peng Y.F., Polizzotti B.D. (2022). Microporous water with high gas solubilities. Nature.

[7] Zhang G.J., Dou L.Z., Xu Y. (2019). Opportunities and challenges of natural gas development and utilization in China. Clean Technol. Environ. Policy.

[8] Sholl D.S., Lively R.P. (2016). Seven chemical separations to change the world. Nature.

[9] Benali M., Aydin B. (2010). Ethane/ethylene and propane/propylene separation in hybrid membrane distillation systems: Optimization and economic analysis. Sep. Purif. Technol..

[10] Timoshenko A., Anokhina E., Akhapkina O. (2016). Energy-Saving Hydrocarbon Distillation with Coupled Heat and Material Flows. Chem. Eng. Technol..

[11] Qiao Z.W., Yan Y.L., Tang Y.X., Liang H., Jiang J.W. (2021). Metal-Organic Frameworks for Xylene Separation: From Computational Screening to Machine Learning. J. Phys. Chem. C.

[12] Wang W.F., Zhang L.L., Cai C.Z., Li S.H., Liang H., Wu Y.F., Zheng H., Qiao Z.W. (2023). Machine learning assisted high-throughput computational screening of MOFs for the capture of chemical warfare agents from the air. Sep. Purif. Technol..

[13] He Y.B., Zhang Z.J., Xiang S.C., Fronczek F.R., Krishna R., Chen B.L. (2012). A robust doubly interpenetrated metal-organic framework constructed from a novel aromatic tricarboxylate for highly selective separation of small hydrocarbons. Chem. Commun..

[14] He Y.B., Xiang S.C., Zhang Z.J., Xiong S.S., Fronczek F.R., Krishna R., O’Keeffe M., Chen B.L. (2012). A microporous lanthanide-tricarboxylate framework with the potential for purification of natural gas. Chem. Commun..

[15] He Y.P., Tan Y.X., Zhang J. (2013). Tuning a layer to a pillared-layer metal-organic framework for adsorption and separation of light hydrocarbons. Chem. Commun..

[16] Zhang M.H., Xin X.L., Xiao Z.Y., Wang R.M., Zhanga L.L., Sun D.F. (2017). A multi-aromatic hydrocarbon unit induced hydrophobic metal-organic framework for efficient C2/C1 hydrocarbon and oil/water separation. J. Mater. Chem. A.

[17] Yuan B.Q., Wang X., Zhou X., Xiao J., Li Z. (2019). Novel room-temperature synthesis of MIL-100(Fe) and its excellent adsorption performances for separation of light hydrocarbons. Chem. Eng. J..

[18] Luo Y., Bag S., Zaremba O., Cierpka A., Andreo J., Wuttke S., Friederich P., Tsotsalas M. (2022). MOF Synthesis Prediction Enabled by Automatic Data Mining and Machine Learning. Angew. Chem. Int. Ed..

[19] Wang Z.H., Zhou T., Sundmacher K. (2022). Interpretable machine learning for accelerating the discovery of metal-organic frameworks for ethane/ethylene separation. Chem. Eng. J..

[20] Demir H., Keskin S. (2023). Revealing acetylene separation performances of anion-pillared MOFs by combining molecular simulations and machine learning. Chem. Eng. J..

[21] Daglar H., Keskin S. (2022). Combining Machine Learning and Molecular Simulations to Unlock Gas Separation Potentials of MOF Membranes and MOF/Polymer MMMs. ACS Appl. Mater. Interfaces.

[22] Wilmer C.E., Leaf M., Lee C.Y., Farha O.K., Hauser B.G., Hupp J.T., Snurr R.Q. (2012). Large-scale screening of hypothetical metal-organic frameworks. Nat. Chem..

[23] Willems T.F., Rycroft C., Kazi M., Meza J.C., Haranczyk M. (2012). Algorithms and tools for high-throughput geometry-based analysis of crystalline porous materials. Microporous Mesoporous Mater..

[24] Dubbeldam D., Calero S., Ellis D.E., Snurr R.Q. (2016). RASPA: Molecular simulation software for adsorption and diffusion in flexible nanoporous materials. Mol. Simul..

[25] Rappe A.K., Casewit C.J., Colwell K.S., Goddard W.A., Skiff W.M. (1992). UFF, a full periodic table force field for molecular mechanics and molecular dynamics simulations. J. Am. Chem. Soc..

[26] Edelsbrunner H., Harer J. (2010). Computational Topology: An Introduction.

[27] Jiang Y., Chen D., Chen X., Li T.Y., Wei G.W., Pan F. (2021). Topological representations of crystalline compounds for the machine-learning prediction of materials properties. NPJ Comput. Mater..

[28] Pun C.S., Lee S.X., Xia K. (2022). Persistent-homology-based machine learning: A survey and a comparative study. Artif. Intell. Rev..

[29] Zomorodian A., Carlsson G. (2004). Computing Persistent Homology. Discret. Comput. Geom..

[30] Ong S.P., Richards W.D., Jain A., Hautier G., Kocher M., Cholia S., Gunter D., Chevrier V.L., Persson K.A., Ceder G. (2013). Python Materials Genomics (pymatgen): A robust, open-source python library for materials analysis. Comput. Mater. Sci..

[31] Tauzin G., Lupo U., Tunstall L., Pérez J.B., Caorsi M., Medina-Mardones A.M., Dassatti A., Hess K. (2021). giotto-tda: A Topological Data Analysis Toolkit for Machine Learning and Data Exploration. J. Mach. Learn Res..

[32] Yuan X.Y., Li L.F., Shi Z.A., Liang H., Li S.H., Qiao Z.W. (2022). Molecular-fingerprint machine-learning-assisted design and prediction for high-performance MOFs for capture of NMHCs from air. Adv. Powder Mater..

[33] Shah M.S., Tsapatsis M., Siepmann J.I. (2016). Identifying Optimal Zeolitic Sorbents for Sweetening of Highly Sour Natural Gas. Angew. Chem. Int. Ed. Engl..

[34] Chen T.Q., Guestrin C., Assoc Comp M. XGBoost: A Scalable Tree Boosting System. Proceedings of the 22nd ACM SIGKDD International Conference on Knowledge Discovery and Data Mining (KDD).

[35] Liang H., Jiang K., Yan T.A., Chen G.H. (2021). XGBoost: An Optimal Machine Learning Model with Just Structural Features to Discover MOF Adsorbents of Xe/Kr. ACS Omega.

[36] Pedregosa F., Varoquaux G., Gramfort A., Michel V., Thirion B., Grisel O., Blondel M., Prettenhofer P., Weiss R., Dubourg V. (2011). Scikit-Learn: Machine Learning in Python. J. Mach. Learn. Res..

[37] Krishnapriyan A.S., Montoya J., Haranczyk M., Hummelshoj J., Morozov D. (2021). Machine learning with persistent homology and chemical word embeddings improves prediction accuracy and interpretability in metal-organic frameworks. Sci. Rep..

[38] Guo S.Y., Huang X.S., Situ Y., Huang Q.H., Guan K.X., Huang J.X., Wang W., Bai X.N., Liu Z.L., Wu Y.F. (2023). Interpretable Machine-Learning and Big Data Mining to Predict Gas Diffusivity in Metal-Organic Frameworks. Adv. Sci..

