# Supplementary material for: Topological Data Analysis Combined with High-Throughput Computational Screening of Hydrophobic Metal–Organic Frameworks: Application to the Adsorptive Separation of C3 Components
Source: Nanomaterials (Basel). 2024 Jan 31;14(3):298. doi: 10.3390/nano14030298 (PMC10857702; doi:10.3390/nano14030298)
Supplement: Supplementary file 1 [file nanomaterials-14-00298-s001.zip › nanomaterials-2835121-supplementary.pdf]

nanomaterials

Supporting Information

Topological Data Analysis Combined with High-Throughput Computational Screening of Hydrophobic Metal–Organic Frameworks: Application to the Adsorptive Separation of C3 Components

Yujuan Yang, Shuya Guo, Shuhua Li, Yufang Wu * and Zhiwei Qiao *

Guangzhou Key Laboratory for New Energy and Green Catalysis, School of Chemistry and Chemical Engineering, Guangzhou University, Guangzhou 510006, China; 2112105114@e.gzhu.edu.cn (Y.Y.); 2112105073@e.gzhu.edu.cn (S.G.); lish@gzhu.edu.cn (S.L.)
* Correspondence: yufang.wu@gzhu.edu.cn (Y.W.); zqiao@gzhu.edu.cn (Z.Q.)

Table of contents:

Lennard-Jones parameters of MOFs. 3
42 Features Extracted from Barcodes 4
Evaluation of XGBoost for S_{C1}, S_{C2}, S_{C3} 5
Lennard-Jones parameters of adsorbates 5
Distribution of computed and predicted data when the XGBoost algorithm predicts *S* and TSN 5
Relative importance values of topological descriptors in the predicted TSN_{C3}.......... 6
Relative importance values of topological descriptors in the predicted S_{C3}.............. 7
Relative importance values of topological descriptors in the predicted N_{C3}............ 8
Topological Features 9
Description of the relevant software used libraries.. 9
Machine Learning 10

Table S1. Lennard-Jones parameters of MOFs.

Atom	ε/k_B [K]	σ [Å]	Atom	ε/k_B [K]	σ [Å]	Atom	ε/k_B [K]	σ [Å]
Ac	16.60	3.10	Ge	190.69	3.81	Po	163.52	4.20
Ag	18.11	2.80	Gd	4.53	3.00	Pr	5.03	3.21
Al	254.09	4.01	H	22.14	2.57	Pt	40.25	2.45
Am	7.04	3.01	Hf	36.23	2.80	Pu	8.05	3.05
Ar	93.08	3.45	Hg	193.71	2.41	Ra	203.27	3.28
As	155.47	3.77	Ho	3.52	3.04	Rb	20.13	3.67
At	142.89	4.23	I	170.57	4.01	Re	33.21	2.63
Au	19.62	2.93	In	301.39	3.98	Rh	26.67	2.61
B	90.57	3.64	Ir	36.73	2.53	Rn	124.78	4.25
Ba	183.15	3.30	K	17.61	3.40	Ru	28.18	2.64
Be	42.77	2.45	Kr	110.69	3.69	S	137.86	3.59
Bi	260.63	3.89	La	8.55	3.14	Sb	225.91	3.94
Bk	6.54	2.97	Li	12.58	2.18	Sc	9.56	2.94
Br	126.29	3.73	Lu	20.63	3.24	Se	146.42	3.75
C	52.83	3.43	Lr	5.53	2.88	Si	202.27	3.83
Ca	119.75	3.03	Md	5.53	2.92	Sm	4.03	3.14
Cd	114.72	2.54	Mg	55.85	2.69	Sn	285.28	3.91
Ce	6.54	3.17	Mn	6.54	2.64	Sr	118.24	3.24
Cf	6.54	2.95	Mo	28.18	2.72	Ta	40.75	2.82
Cl	114.21	3.52	N	34.72	3.26	Tb	3.52	3.07
Cm	6.54	2.96	Na	15.09	2.66	Tc	24.15	2.67
Co	7.04	2.56	Ne	21.13	2.66	Te	200.25	3.98
Cr	7.55	2.69	Nb	29.69	2.82	Th	13.08	3.03
Cu	2.52	3.11	Nd	5.03	3.18	Ti	8.55	2.83
Cs	22.64	4.02	No	5.53	2.89	TI	342.14	3.87
Dy	3.52	3.05	Ni	7.55	2.52	Tm	3.02	3.01
Eu	4.03	3.11	Np	9.56	3.05	U	11.07	3.02
Er	3.52	3.02	O	30.19	3.12	V	8.05	2.80
Es	6.04	2.94	Os	18.62	2.78	W	33.71	2.73
F	25.16	3.00	P	153.46	3.69	Xe	167.04	3.92
Fe	6.54	2.59	Pa	11.07	3.05	Y	36.23	2.98
Fm	6.04	2.93	Pb	333.59	3.83	Yb	114.72	2.99
Fr	25.16	4.37	Pd	24.15	2.58	Zn	62.39	2.46
Ga	208.81	3.90	Pm	4.53	3.16	Zr	34.72	2.78

Table S2. 42 Features Extracted from Barcodes.

No	Feature vector
0	0D_the number of birth-death pair
1	0D_total death time
2	0D_the maximum gap between death times
3	0D_mean death time
4	0D_std death time
5	0D_the maximum death time
6	0D_the minimum death time
7	0D_the average death times
8	0D_the number of birth-death pair types
9	1D_the number of birth-death pair
10	1D_total survival time
11	1D_mean survival time
12	1D_std survival time
13	1D_the maximum survival time
14	1D_the minimum survival time
15	1D_mean birth time
16	1D_std birth time
17	1D_the maximum birth time
18	1D_the maximum gap between birth times
19	1D_mean death time
20	1D_std death time
21	1D_the maximum death time
22	1D_the minimum death time
23	1D_the average death times

24	1D_the average birth times
25	2D_the number of birth-death pair
26	2D_the maximum gap between birth times
27	2D_total survival time
28	2D_mean survival time
29	2D_std survival time
30	2D_the maximum survival time
31	2D_the minimum survival time
32	2D_mean birth time
33	2D_std birth time
34	2D_the maximum birth time
35	2D_the minimum birth time
36	2D_mean death time
37	2D_std death time
38	2D_the maximum death time
39	2D_the minimum death time
40	2D_the average death times
41	2D_the average birth times

Table S3. Evaluation of XGBoost for S_{C1}, S_{C2}, S_{C3}.

Performance	R^2 Scores				RMSE			
	Structural	Topological	T + S	Δ	Structural	Topological	T + S	Δ
S_{C1}	0.227	0.216	**0.253**	11.45%	2.396	2.413	**2.355**	1.70%
S_{C2}	0.468	0.500	**0.667**	42.52%	0.442	0.429	**0.350**	20.89%
S_{C3}	0.720	0.730	**0.804**	11.67%	3.282	3.224	**2.747**	16.31%

Table S4. Lennard-Jones parameters of adsorbates.

Atom	ε/k_B [K]	σ [Å]
CH_4_sp3	148.0	3.75
CH_3_sp3	98.0	3.75
CH_2_sp3	46.0	3.95

Distribution of computed and predicted data when the XGBoost algorithm predicts *S* and TSN

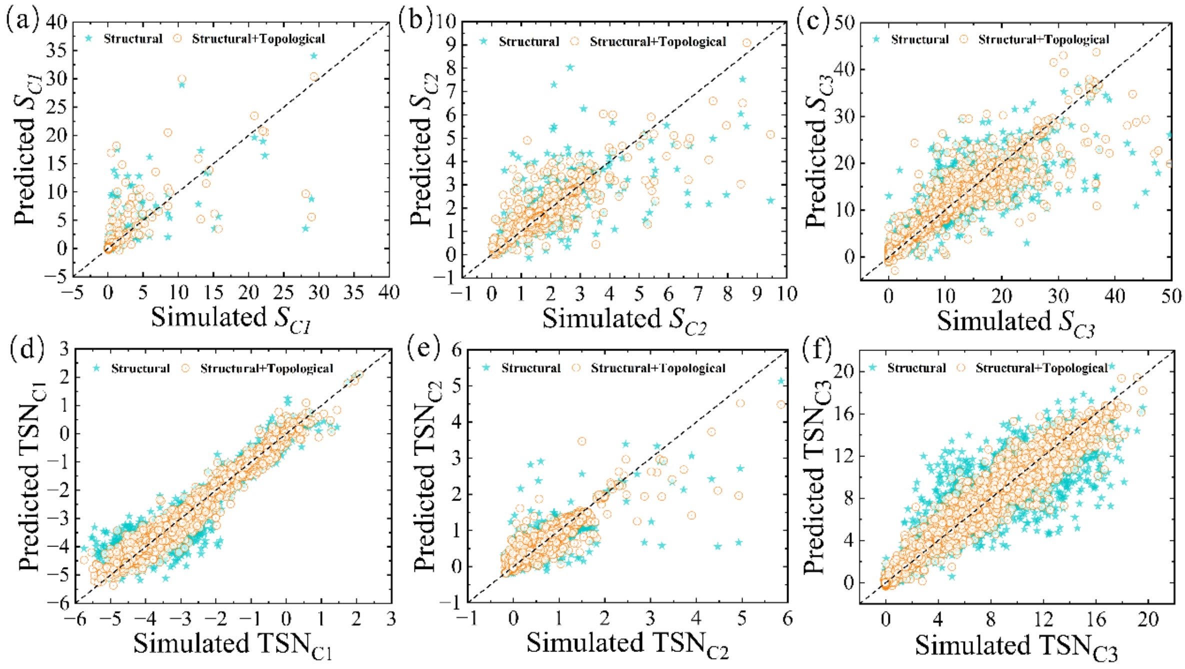

Figure S1. Distribution of computed and predicted data when the XGBoost algorithm predicts (a)S_{C1}, (b)S_{C2}, (c)S_{C3}, (d)TSN_{C1}, (e)TSN_{C2}, and (f)TSN_{C3} using different combinations of features.

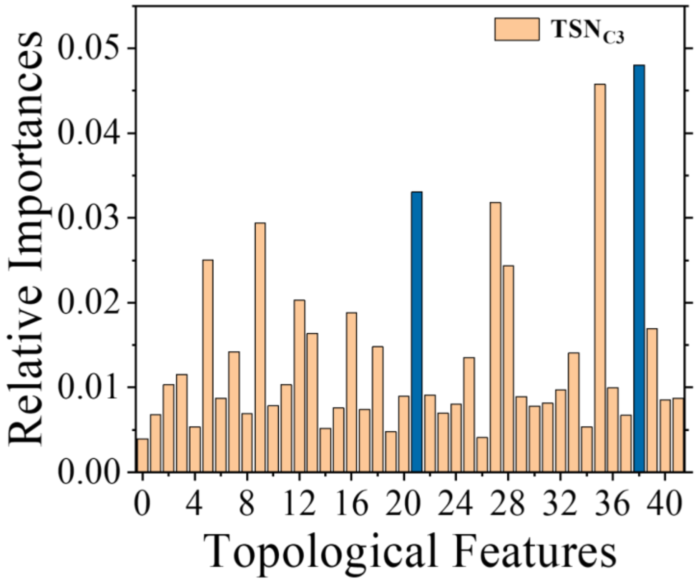

Figure S2. Relative importance values of topological descriptors in the predicted TSN_{C3} (blue bars are the most important descriptors among 1D and 2D topological descriptors).

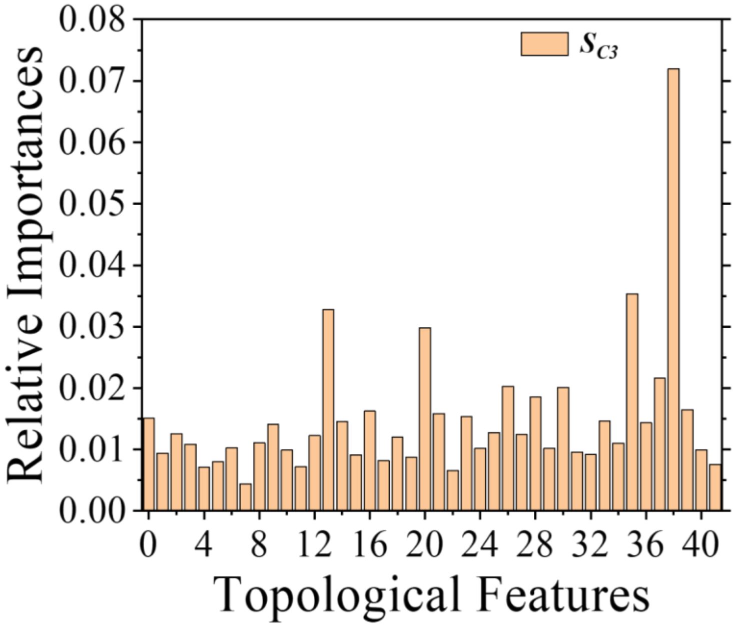

Figure S3. Relative importance of the topological descriptor in predicting S_{C3}.

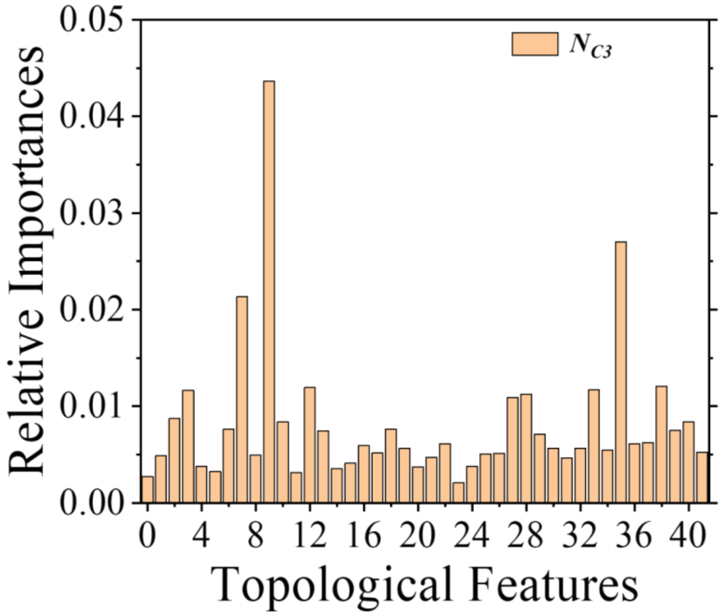

Figure S4. Relative importance of the topological descriptor in predicting N_{C3}.

Topological Features

Structural properties that remain unchanged under continuous transformations (or topological transformations) are called shape features (topological features). And similar structural properties exist within complex high-dimensional data, which we can figuratively call the shape (features) of the data. Topological Data Analysis (TDA) is a topology-based data analysis method that captures the structure and shape of data under different data representations. As a powerful data analysis tool, TDA can efficiently capture topological information of high-level data space and is widely used in various fields related to big data.

Description of the relevant software libraries used Pymatgen

Pymatgen (Python Materials Genomics) is an API package that connects to materials projects for high-throughput computations, one of the most powerful Python packages for performing high-throughput materials computations, and is a program developed and maintained by Shyue Ping Ong, Professor of Nanoengineering at the University of California, San Diego Jacobs School of Engineering, and his team at the Materials Virtual Lab. Ong and his team at the Materials Virtual Lab develop and maintain the software. Here are some of the key features:

Highly flexible classes for the representation of Element, Site, Molecule, and Structure objects.

Extensive input/output support, including support for VASP, ABINIT, CIF, Gaussian, XYZ, and many other file formats.

Powerful analysis tools, including generation of phase diagrams, Pourbaix diagrams, diffusion analyses, reactions, etc.

Electronic structure analyses, such as density of states and band structure.

Integration with the Materials Project REST API, Crystallography Open Database, and other external data sources.

Giotto-tda

Giotto-tda is a high-performance topological machine learning toolbox in Python built on top of scikit-learn and is distributed under the GNU AGPLv3 license. It is part of the Giotto family of open-source projects. The functionalities of giotto-tda are provided in scikit-learn-style transformers. This allows you to generate topological features from your data in a familiar way.

Machine Learning

The formula for the calculation of the model evaluation indicators

RMSE is one of the common measures of model prediction error that calculates the difference between predicted and actual values and converts it to a measure of standard deviation. A lower RMSE indicates that the model fits the actual data better because it measures the average error between the model's predicted and actual values. As the RMSE decreases, it indicates that the model's predictions are closer to the actual observations, i.e. the model is performing better. R^2 is a commonly used metric for evaluating regression models that indicate the model's ability to explain the variance in the observed data. The value of R^2 ranges from 0 to 1, with the value of R^2 closer to 1 indicating that the model explains the variance in the observed data better.

$$\text{RMSE} = \sqrt{\frac{1}{n}\sum_{i=1}^{n}(\hat{y_i} - y_i)^2} \quad (1)$$

$$R^2 = 1 - \frac{\sum_{i=1}^{n}(y_i - \hat{y_i})^2}{\sum_{i=1}^{n}(y_i - \bar{y})^2} \quad (2)$$

where n、y_i、$\hat{y_i}$、$\bar{y}$ represents the number of MOFs, the simulated values, the predicted values and the predicted mean values.

XGBoost Algorithm

Extreme Gradient Boosting (XGBoost) is a gradient-boosting algorithm that belongs to the category of reinforcement learning. It has been very successful in solving various data science problems, especially with structured data. Below are the main features of the XGBoost algorithm:

Gradient boosting framework: XGBoost is a gradient boosting algorithm that iteratively trains multiple weak learners (usually gradient decision trees), where the learning objective of each round is to fit the residuals of the previous round.

Regularisation: XGBoost introduces regularisation terms, including L1 (lasso) and L2 (ridge) regularisation, to avoid overfitting. This helps to improve the generalization ability of the model.

Column sampling: XGBoost supports feature sub-sampling, which samples features in each iteration, helping to improve model diversity and reduce the risk of over-fitting.

Parallel processing: XGBoost is capable of parallel computation, which improves model training efficiency for large datasets through parallel processing.

Missing value processing: XGBoost can automatically handle missing values without additional processing by the user.

Automatic pruning: XGBoost uses pre-pruning and post-pruning strategies to avoid generating overly complex trees.

In this work, the hyperparameters used in the XGBoost algorithm are: n_esti mators=1000, max_depth=7, eta=0.1, subsample=0.7, colsample_bytree=0.8.
